# Direct Cell-Cell Contact between Mesenchymal Stem Cells and Endothelial Progenitor Cells Induces a Pericyte-Like Phenotype In Vitro

**DOI:** 10.1155/2014/395781

**Published:** 2014-01-20

**Authors:** Markus Loibl, Andreas Binder, Marietta Herrmann, Fabian Duttenhoefer, R. Geoff Richards, Michael Nerlich, Mauro Alini, Sophie Verrier

**Affiliations:** ^1^AO Research Institute Davos, Clavadelerstrasse 8, 7270 Davos, Switzerland; ^2^Department of Trauma Surgery, Regensburg University Medical Center, 93042 Regensburg, Germany; ^3^Department of Oral and Maxillofacial Surgery, Albert-Ludwigs-University, 79106 Freiburg, Germany

## Abstract

Tissue engineering techniques for the regeneration of large bone defects require sufficient vascularisation of the applied constructs to ensure a sufficient supply of oxygen and nutrients. In our previous work, prevascularised 3D scaffolds have been successfully established by coculture of bone marrow derived stem cells (MSCs) and endothelial progenitor cells (EPCs). We identified stabilising pericytes (PCs) as part of newly formed capillary-like structures. In the present study, we report preliminary data on the interactions between MSCs and EPCs, leading to the differentiation of pericyte-like cells. MSCs and EPCs were seeded in transwell cultures, direct cocultures, and single cultures. Cells were cultured for 10 days in IMDM 10% FCS or IMDM 5% FCS 5% platelet lysate medium. Gene expression of PC markers, CD146, NG2, **α**SMA, and PDGFR-**β**, was analysed using RT-PCR at days 0, 3, 7, and 10. The upregulation of CD146, NG2, and **α**SMA in MSCs in direct coculture with EPCs advocates the MSCs' differentiation towards a pericyte-like phenotype in vitro. These results suggest that pericyte-like cells derive from MSCs and that cell-cell contact with EPCs is an important factor for this differentiation process. These findings emphasise the concept of coculture strategies to promote angiogenesis for cell-based tissue engineered bone grafts.

## 1. Introduction

The remarkable regeneration potential of bone tissue is based on the presence of a highly branched vessel network, providing oxygen, nutrients, growth factors, and precursor cells to the injured tissue [1]. However, the capacity of regeneration is limited and fails in large bone defects. Thus, the gold-standard strategy remains autologous bone implants, associated with an additional surgical procedure at the harvesting site causing an increase of operation time, pain, and risk for infection at the donor site.

Cell-based tissue engineering strategies have been developed and are accepted since a few years to address the challenge of bone repair. The approach of engineering bone tissue not only depends on the presence of osteogenic cells at the healing site but also requires an adequate vascularisation of the applied biomaterial [2]. Therefore, bone-forming osteoblasts and endothelial cells (or their progenitors) are playing a crucial role for successful engraftment of cell seeded biomaterials [3].

Bone marrow is a natural reservoir of mesenchymal stem cells (MSCs) able to develop an osteogenic, chondrogenic, and adipogenic phenotype upon stimulation. The recruitment, growth, and differentiation of MSCs into mature osteoblasts are regulated by many cytokines and growth factors. These factors are secreted not only by the osteoblasts themselves, but also from endothelial cells from the tightly connected vascular network [2, 4, 5]. This crosstalk between different cell types is enabled by secreted paracrine factors, as well as by direct cell-to-cell interactions.

In 1997, Asahara et al. identified the presence of bone marrow derived endothelial progenitor cells (EPCs) able to develop an endothelial phenotype in peripheral blood of adults. Those progenitor cells are known to express CD34 [6], CD133 [7], and CD309 [8] and as mature endothelial cells, they specifically bind UEA-1 lectins [7, 9]. As they are present in the blood compartment as well as in the bone marrow, they can easily be isolated in an autologous way, which is a fundamental advantage for further tissue engineering applications.

Recently, we developed an in vitro prevascularised 3D polyurethane (PU) bone implant seeded with EPCs (CD34+/CD133+) and MSCs, which showed the formation of luminal tubular structures after 7 days of coculture [10]. These capillary-like structures expressed not only mature endothelial cell markers (i.e., PECAM-1, vWF), but also pericyte (PC) markers (i.e., CD146, NG2, and *α*SMA). PCs are still poorly characterised [11] and their reported markers are also expressed on other cells associated with blood vessels [11, 12]. Coexpression of several markers (CD146 [13, 14], NG2 [15], *α*SMA [16], and PDGFR-*β* [17]) and absence of endothelial markers (PECAM-1) [7] may be used to identify PCs in vitro. However, coexpression of these markers by PCs is variable and depends not only on the tissue of origin [18], but also on culture conditions [19]. Hellström et al. [20] and more recently Blocki et al. suggested that PCs represent a subpopulation of MSCs in bone marrow, contributing to microvessel maturation, stability, structure, and function [19, 20]. PCs establish important direct cell-cell contact with endothelial cells of immature blood vessels [21] and some studies suggested that PCs may serve as guiding structures aiding outgrowth of endothelial cells to form early capillary sprouts [22].

Based on our previous findings concerning the participation of PC-like cells in the overall structure of in vitro preformed capillary network [10], the aim of the present study was to determine the origin of PC-like cells in cocultures of MSCs and EPCs in vitro. We analysed the influence of cell-cell interactions in two different 2D coculture systems. In addition, two cell culture media were tested; on one hand, cells were cultured in a classical cell culture medium without any growth factor supplements; on the other hand, the medium was supplemented with autologous platelet lysate growth factors (PL) to provide the optimal condition for EPCs [23].

## 2. Materials and Methods

### 2.1. Preparation of Platelet Lysate Growth Factors (PL)

PL was prepared from platelet concentrates, as described earlier [23]. Platelet bags were obtained from the blood bank of Kantonsspital Graubünden in Chur in accordance with the current ethical laws of Switzerland. The platelet bags contained a standardised platelet density (5 times above physiological concentration), obtained by blood apheresis. The platelet density was further increased by a centrifugation at 2000 g for 20 minutes. After two washing steps in phosphate buffer saline (PBS) and subsequent centrifugation, the platelet pellets were resuspended in half of the original volume of PBS to obtain a final density 10 times higher than that in normal blood (2.5 million (±10%) platelets/*μ*L). PL samples were pooled from platelet concentrates from three different donors and randomly matched.

### 2.2. Bone Marrow Aspirates

Human bone marrow aspirates (20 mL) were obtained upon informed consent and ethical approval (EK Regensburg 12-101-0127) from 6 donors undergoing routine orthopaedic surgery (20 to 76 years old, average age 43 years; 4 females and 2 males). The samples were processed within 24 hours after harvesting.

### 2.3. Mesenchymal Stem Cells (MSC)

Bone marrow mononucleated cells (BMCs) were isolated from bone marrow aspirates by Histopaque-1077 (Sigma-Aldrich) density gradient centrifugation, as previously described [24]. Briefly, bone marrow was homogenised and diluted 1 : 4 with PBS. After slowly pipetting on Histopaque, the samples were centrifuged at 800 g for 20 minutes. The interphase containing BMCs was collected and washed twice in Iscove Modified Dulbecco Medium (IMDM, Gibco) containing 10% human MSC qualified fetal calf serum (FCS, Gibco), followed by 15 min centrifugation at 400 g. BMCs were seeded at the density of 16 × 10^6^ mononucleated cells per 300 cm^2^ cell culture flask (BD Biosciences, Falcon Cell Culture Dishes) and cultured in Minimum Essential Medium Alpha (MEM*α*, Gibco) containing PenStrep (PS) (100 U/mL, Gibco), 10% FCS, and 5 ng/mL basic fibroblast growth factor (bFGF) (R&D Systems). After the first passage, adhesion selected MSCs were further subcultured at a density of 0.9 × 10^6^ per 300 cm^2^ cell culture flask.

### 2.4. Endothelial Progenitor Cells (EPC) and CD34-CD133-CD146-Depleted MSCs (Depleted-MSCs)

CD34+, CD133+, and CD146+ cell populations were further selected from the previously isolated BMCs using magnetic microbeads linked to antibodies specific to CD133, CD34, or CD146 (MACS system, Miltenyi Biotec) according to the manufacturer instructions. After isolation, the different cell populations were referred to as CD133+, CD34+, or CD146+, respectively. The remaining cell population (depleted from CD133+, CD34+, and CD146+ cells) was termed depleted-MSCs. CD34+ and CD133+ cells were pooled and from then on referred to as EPCs. Cells from the depleted-MSCs population were propagated in MEM*α* containing PS (100 U/mL), 10% FCS, and 5 ng/mL bFGF. EPCs were cultured in IMDM containing PS (100 U/mL), supplemented with 5% PL, 5% FCS, and 1% nonessential amino acids (NEAA, Gibco). All cell types were cultured at 37°C 5% CO_2_ humidified atmosphere incubator and media were changed twice a week.

### 2.5. Cell Culture

**Figure 1:**
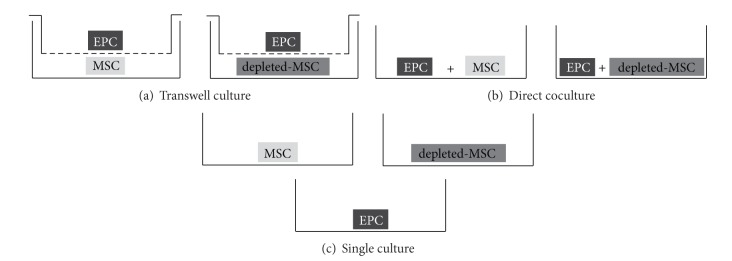
Cell culture setups. MSCs or depleted-MSCs were seeded with EPCs in transwell culture (a) or direct coculture (b). MSCs, depleted-MSCs, and EPCs were seeded in single cultures (c) as controls. All experiments were performed in the presence of IMDM-FCS (IMDM supplemented with 10% FCS) or IMDM-PL (IMDM supplemented with 5% FCS, 5% PL).

For all subsequent experiments, cells between passages 2 and 3 were used. MSCs, depleted-MSCs, and EPCs (CD34+/CD133+) were enzymatically detached (Trypsin-EDTA), counted, seeded at a density of 5,000 cells/cm^2^ in 3 different culture setups (Figure 1), and incubated for 3, 7, or 10 days in presence of IMDM-FCS (IMDM supplemented with 10% FCS and 1% NEAA) or IMDM-PL (IMDM supplemented with 5% FCS, 5% PL, and 1% NEAA).

For indirect cocultures (transwell), MSCs (or depleted-MSCs) were seeded in the bottom part of 6-well plates at a density of 5,000 cells/cm^2^ for transwell culture setup (Figure 1(a)), whereas EPCs were seeded at the same density in the corresponding transwell cell culture inserts (0.4 *μ*m pore size, Sigma-Aldrich).

For direct coculture experiments (direct coculture), MSCs and EPCs, as well as depleted-MSCs and EPCs, were coseeded in a 1 : 1 ratio in 75 cm^2^ cell culture plate (BD Biosciences) at an initial density of 5,000 cells/cm^2^ (Figure 1(b)). In addition, each cell type was seeded individually in 6-well plates (5,000 cells/cm^2^) for single culture as controls (Figure 1(c)).

### 2.6. Cell Sorting

Prior to gene expression analysis, cells in direct cocultures were separated by fluorescence activated cell sorting (FACS) using FITC labelled lectin (UEA-1, Sigma-Aldrich) which specifically labels EPCs. Adherent cells were incubated for 60 minutes at 37°C in the dark with 10 *μ*g/mL FITC-lectin in IMDM-FCS as previously described [7]. Afterwards, cells were washed three times with PBS and trypsinised, followed by centrifugation at 2000 g for 10 min. The resulting cell pellet was resuspended in 500 *μ*L PBS 1% FCS, filtered through a 40 *μ*m cell strainer, and subjected to FACS analysis (FACSAria III, BD Biosciences). FITC signal was identified with a 488 nm excitation laser and 502LP filter. Cell analysis was performed on at least 10,000 events for each sample and analysed using FACS DIVA software version 6.1.3 (BD Biosciences). A primary gate based on physical parameters (forward and side scatter) was set to exclude dead cells or small debris. For the separation, gating was implemented based on the negative-control staining profile of MSCs in direct coculture with EPCs. Sorting gates were determined by fluorescence intensity of FITC maintaining a gap between the lectin negative and the lectin positive gate. Cells were fractionated with an efficiency of >90% in lectin positive and lectin negative cell populations and collected in PBS 1% FCS. The lectin positive cell population represents EPCs, and the lectin negative population represents MSCs or depleted-MSCs. At least 50,000 events per sorted cell type were collected. After separation, cells were spin down and mRNA was extracted for further analysis. In parallel, the sorted cell populations were reseeded (1 : 1 ratio) onto a 2-well glass slide (LabTek chamber slides, Thermo Fisher scientific) at 5,000 cells per cm^2^ for immunofluorescence staining.

### 2.7. Real-Time PCR

At days 3, 7, and 10, total RNA was extracted from monolayers or sorted cells using Tri Reagent (Molecular Research Centre, Inc.) according to the manufacturer's protocol and stored at −80°C until further use. cDNA was synthesised from 1 *μ*g RNA using TaqMan reverse transcription reagents (Applied Biosystems, Invitrogen) with MultiScribe reverse transcriptase (50 U/*μ*L) and random hexamer primers. Real-time Polymerase chain reaction (PCR) was performed on the StepOne Plus machine (Applied Biosystems). Genes of interest were detected using CD146/MCAM (HS00174838_m1), NG2/CSPG4 (HS00426981_m1), *α*SMA/ACTA2 (HS00909449_m1), PECAM-I (HS01065282_m1), and PDGFR-*β* (HS00182163_m1) all purchased from Applied Biosystems. Human GAPDH (Cat no. 4326317E, Applied Biosystems) was used as a housekeeping gene. PCR conditions were 95°C for 10 min, followed by 45 cycles of amplification at 95°C for 15 sec and 60°C for 1 min using the Stepone software v2.1 (Applied Biosystems). Relative quantification of mRNA targets was performed according to the comparative ΔΔCt method.

### 2.8. Immunocytochemistry

After 7 days of cell culture in IMDM-FCS or IMDM-PL, cells were separated and seeded onto LabTek chamber slides. Cells were allowed to adhere for at least 6 hours prior to fixation with 70% methanol for 10 minutes and 100% methanol for 2 minutes and stored at −20°C until further use. Cells were rehydrated in PBS prior to staining procedure. Nonspecific bindings were blocked by incubation with 5% normal goat serum (Vector Labs) in PBS for 60 minutes. Thereafter, slides were incubated with a mouse anti-human antibody (anti-hCD146 (clone P1H12, ab24577) or anti-hNG2 (clone LHM2, ab20156)) and a rabbit anti-human antibody (anti-hNG2 (ab104535) or anti-h*α*SMA (ab5694)) simultaneously. Mouse anti-human and rabbit anti-human antibodies were used in 3 different combinations: CD146/NG2, CD146/*α*SMA, and NG2/*α*SMA. After 1 h of incubation at room temperature, the samples were washed and stained with corresponding secondary fluorescent antibodies: Alexa Fluor 488 conjugated anti-mouse antibody (Life Technologies, A11029) and TRITC conjugated anti-rabbit antibody (abcam, ab50598).

Primary and secondary antibodies were both incubated for 1 hour at room temperature. Stained slides were cover-slipped with Prolong Gold Antifade reagent with DAPI (Molecular Probes, Life Technologies). A digital image was obtained by using an AxioCam HRc camera and AxioVision software V3.1 (Carl Zeiss).

### 2.9. Statistical Analysis

Results are presented as mean ± standard error of the mean. Statistical analysis was performed using Prism 4 software (Graphpad). One-way ANOVA with Tukey's multiple comparison test was used to calculate the overall differences in nonsize matched experimental groups. Level of significance was *P* < 0.05.

## 3. Results

### 3.1. Cell Characterisation

**Figure 2:**
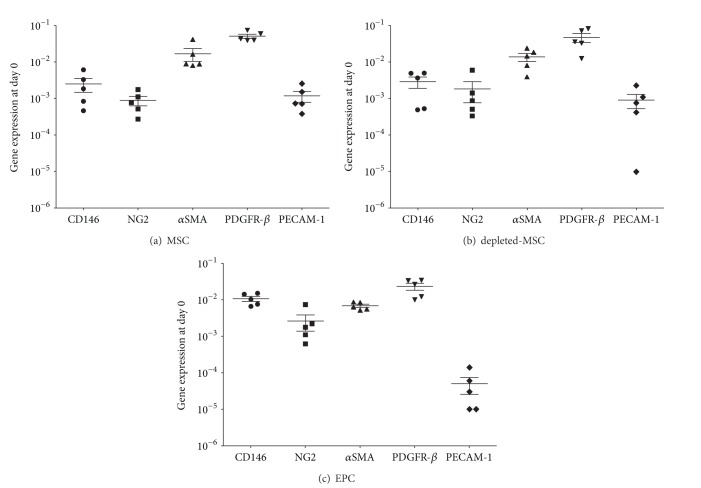
Baseline gene expression at day 0 of MSCs, depleted-MSCs, and EPCs (ΔCt values relative to GAPDH in logarithmic scale). Pericyte marker genes (CD146, NG2, *α*SMA, and PDGFR-*β*) and an endothelial marker gene (PECAM-1) were detected in MSCs (a), depleted-MSCs (b), and EPCs (c) after single culture in culture medium at day 0 in 5 independent experiments.

The baseline expression of the pericyte and endothelial marker genes in each of the 3 tested cell populations (MSCs, depleted-MSCs, and EPCs) was measured prior to exposure to the different culture conditions (day 0) using GAPDH as a housekeeping gene. Similar expression profile was observed on day 0 for the MSCs and depleted-MSCs populations when propagated in MEM*α* supplemented with 10% FCS and 5 ng/mL bFGF (Figures 2(a) and 2(b)). Moreover, *α*SMA and PDGFR-*β* were expressed at a higher level compared to CD146 and NG2 in MSCs and depleted-MSCs, without a significant difference (*P* > 0.05). However, as expected PECAM-1 was expressed at a lower level than the PC marker genes.

A similar trend of baseline gene expression was observed for EPCs population after culturing in IMDM supplemented with 5% FCS and 5% PL, without a significant difference for all tested genes (*P* > 0.05). The endothelial marker PECAM-1 was expressed at a lower level than the PC marker genes similar to MSCs and depleted-MSCs.

### 3.2. Influence of Cell Culture Setups on Gene Expression

**Figure 3:**
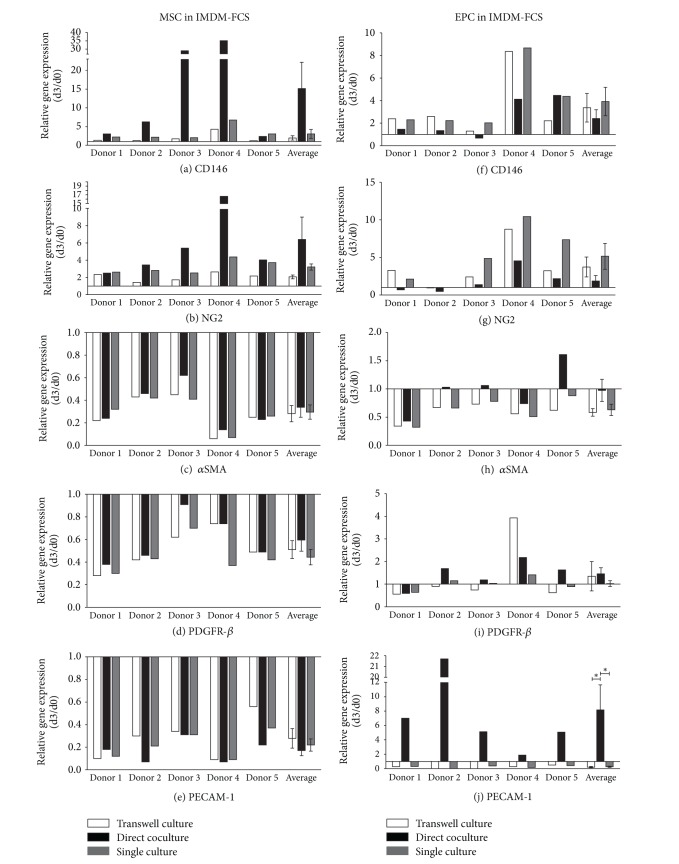
Gene regulation in MSCs and EPCs after 3 days of culture in IMDM-FCS. Pericyte marker genes CD146 ((a), (f)), NG2 ((b), (g)), *α*SMA ((c), (h)), and PDGFR-*β* ((d), (i)) and endothelial marker gene PECAM-1 ((e), (j)) were detected in MSCs and EPCs after 3 days in transwell culture, direct coculture, and single culture in IMDM-FCS (IMDM supplemented with 10% FCS). Results are presented for each gene as relative change in gene expression (2^−ΔΔCt^) over time between day 0 and day 3 in 5 independent experiments (donor 1–5) and as average ± error of the mean of all experiments.

Gene expression analysis of MSCs cultured in IMDM-FCS revealed an upregulation of CD146 (Figure 3(a)) for all time points and all cell media when compared to day 0. Representative data are shown for day 3 and are summarised in Supplementary Table  1 (see Supplementary Material available online at http://dx.doi.org/10.1155/2014/395781). CD146 expression demonstrated a 15.1 ± 6.99-fold increase compared to day 0 in coculture and a 3.25 ± 0.89- and 1.97 ± 0.58-fold increase in single and transwell culture, respectively (Figure 3(a)). A similar trend was detected for NG2 but to a lesser extent. NG2 expression demonstrated a 6.4 ± 2.6-fold increase compared to day 0 in coculture and a 3.21 ± 0.36- and 2.06 ± 0.21-fold increases in single and transwell culture, respectively (Figure 3(b)). An overall decrease of *α*SMA and PDGFR-*β* expression was observed for MSCs, with a lower extent for the direct coculture condition (Figures 3(c) and 3(d)). The expression of PECAM-1 was decreased in MSC in all culture conditions and at all time points (Figure 3(e)). A similar trend of gene expression was shown for depleted-MSCs (Supplementary Figure  1).

Analysis of EPCs revealed an increased CD146 expression in all culture setups. In contrast to MSC, direct coculture induced the lowest level of upregulation (2^−ΔΔCt^2.42 ± 0.78, Figure 3(f)). A similar trend was apparent for NG2 but to a lesser extent (Figure 3(g)).

The *α*SMA expression showed a constant decrease in single culture or transwell culture (2^−ΔΔCt^0.63 ± 0.1 and 2^−ΔΔCt^0.58 ± 0.07, resp.). However, EPCs in direct coculture with MSC demonstrated no change in *α*SMA expression (2^−ΔΔCt^0.97 ± 0.2) (Figure 3(h)). The PDGFR-*β* expression showed a marginal increase in direct coculture with MSCs (2^−ΔΔCt^1.46 ± 0.27) (except donor 1), a minor increase in transwell culture (2^−ΔΔCt^1.35 ± 0.65) (except donor 4), and no changes in gene expression in single culture (Figure 3(i)).

Of interest, a significant upregulation of PECAM-1 was observed in EPCs (2^−ΔΔCt^8.16 ± 3.49) when cocultured in direct cell-cell contact with MSCs in comparison to single or transwell culture (2^−ΔΔCt^0.26 ± 0.08 and 2^−ΔΔCt^0.21 ± 0.09) (Figure 3(j)).

EPC gene expression in all culture setups revealed similar trends when EPCs were cocultured with depleted-MSCs (Supplementary Figure  1).

### 3.3. Influence of Culture Medium on Gene Expression

**Figure 4:**
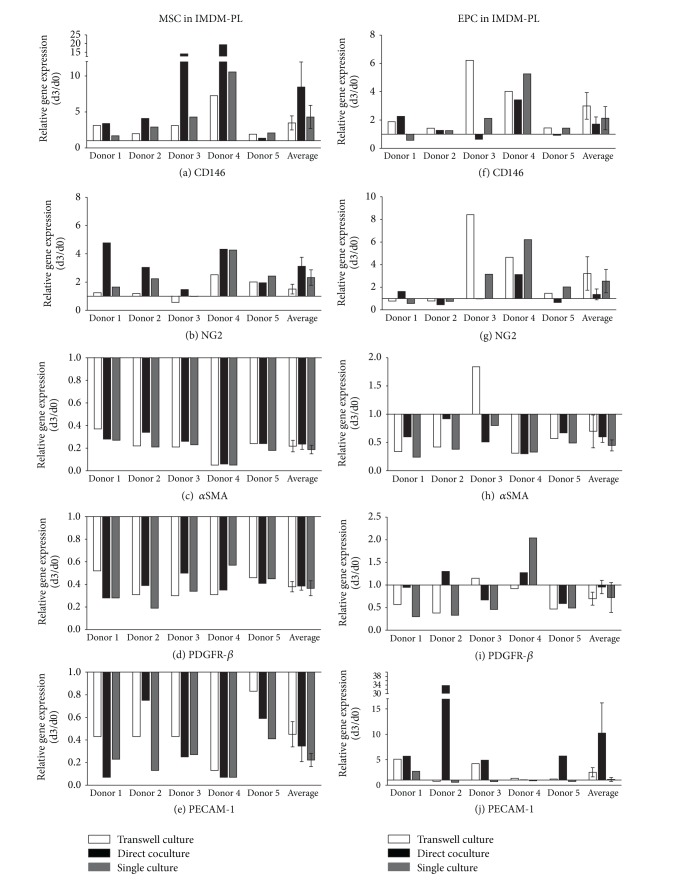
Gene regulation in MSCs and EPCs after 3 days of culture in IMDM-PL. Pericyte marker genes CD146 ((a), (f)), NG2 ((b), (g)), *α*SMA ((c), (h)), and PDGFR-*β* ((d), (i)) and endothelial marker gene PECAM-1 ((e), (j)) were detected in MSCs and EPCs after 3 days in transwell culture, direct coculture, and single culture in IMDM-PL (IMDM supplemented with 5% FCS and 5% PL). Results are presented for each gene as relative change in gene expression (2^−ΔΔCt^) over time between day 0 and day 3 in 5 independent experiments (donor 1–5) and as average ± error of the mean of all experiments.

Gene expression analysis of MSCs, depleted-MSCs, and EPCs cultured in IMDM-PL (Figure 4 and Supplementary Figure  2) revealed the same trend of changes as for IMDM-FCS at all time points. Representative data are presented for day 3 (Figure 4 and Supplementary Figure  2). However, the differences between the cell culture conditions were less prominent as detected for IMDM-FCS medium. Similar to IMDM-FCS, highest upregulation of CD146 and NG2 expression was detected in MSCs in direct coculture with EPCs (Figures 4(a) and 4(b)). CD146 expression demonstrated a 8.44 ± 3.5-fold increase in direct coculture and a 4.3 ± 1.62- and 3.47 ± 0.98-fold increases in single and transwell culture, respectively (Figure 4(a)). In addition, NG2 expression revealed a 3.11 ± 0.64-fold increase for cells in direct coculture, a 2.32 ± 0.55-fold increase for single culture, and a 1.51 ± 0.34-fold increase for transwell culture (Figure 4(b)).

An overall decrease in gene expression of *α*SMA and PDGFR-*β* was observed in MSCs after 3 days when cultured in IMDM FCS (Figures 4(c) and 4(d)). The 2^−ΔΔCt^ values (day 3/day 0) of PECAM-1 decreased in MSCs for all conditions (Figure 4(e)).

EPCs in IMDM-PL showed an upregulation of CD146 and NG2 at day 3 in all culture setups. In contrast to MSCs, EPCs showed the lowest upregulation in direct coculture with MSCs in comparison to single or transwell cultures. In detail, CD146 expression in EPCs direct coculture with MSCs propagated in IMDM-PL showed a 1.7 ± 0.51-fold increase compared to a 2.13 ± 0.82- and 3.0 ± 0.94-fold increases in single or transwell culture, respectively (Figure 4(f)). A 1.36 ± 0.48-fold increase was observed for NG2 expression in EPCs in coculture with MSCs in IMDM-PL, and a 2.54 ± 1.03- and 3.22 ± 1.48-fold upregulation in single and transwell culture were observed, respectively (Figure 4(g)).

Gene expression of *α*SMA and PDGFR-*β* in EPCs was likewise not affected by the different cell-culture setups. Both genes were downregulated at day 3 without significant differences between culture conditions (Figures 4(h) and 4(i)).

Noteworthy, a 10.2 ± 5.94-fold increase of PECAM-1 was observed in EPCs in direct coculture with MSCs in IMDM-PL. PECAM-1 expression of EPCs was of lesser extent in single and transwell culture, with a 1.12 ± 0.40- and 2.53 ± 0.89-fold increase, respectively (both > 0.05) (Figure 4(j)).

### 3.4. MSCs in Direct Coculture Coexpress CD146, *α*SMA, and NG2

**Figure 5:**
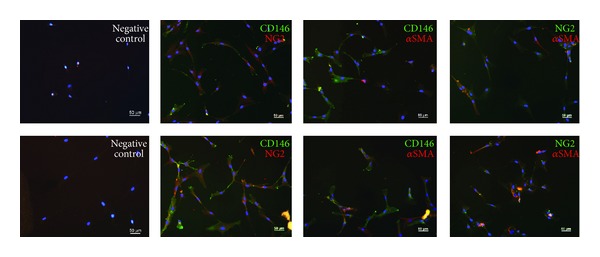
Coexpression of pericyte markers on MSCs after direct coculture with EPCs for 7 days in IMDM-FCS (top row) or IMDM-PL (bottom row). Immunofluorescence staining for CD146, NG2, and *α*SMA demonstrated double positive cells for each combination (CD146 and NG2, CD146 and *α*SMA, and NG2 and *α*SMA). Negative controls were stained only with the fluorescent labelled secondary antibody. Cell nuclei are stained with DAPI (blue). Scale bars = 50 *μ*m.

Expression of the PC markers CD146, *α*SMA, and NG2, were detected by immunofluorescence staining on MSCs after 7 days when cocultured with EPCs and consecutive separation. Single positive and double positive cells were detectable, in both IMDM-FCS and IMDM-PL (Figure 5).

## 4. Discussion

The aim of the present study was to investigate the interactions between EPCs and MSCs in cocultures to determine the origin of PC-like cells in vitro. In addition, we evaluated the influence of platelet derived growth factors on EPCs and MSCs in this experimental setup.

In our previous work, we successfully established a 3D prevascularised scaffold in vitro [10]. We demonstrated a beneficial effect of MSCs coseeded with EPCs and observed the formation of luminal tubular structures already after 7 days in vitro. Notably, tubular structures were positive for CD146, NG2, and *α*SMA, suggesting the presence of PC-like cells.

In the present study, MSCs and EPCs were cultured in direct contact or in a transwell system to elucidate the influence of soluble, paracrine factors and to identify the population of origin of in vitro differentiated PCs.

It is well accepted that CD34 and CD133 are EPC markers [6]. In contrast to EPC, PCs do not express CD34 and CD133; however, they do express CD146 [13, 14, 25]. Therefore, we assume that the depleted-MSC cell population, characterised by the absence of CD34, CD133, or CD146, is free of EPCs and CD146+ PCs.

This was confirmed by gene expression analysis of depleted-MSCs at day 0, which was similar to the profile obtained for MSCs.

To date, there is no single surface marker that is specific or exclusively expressed on PCs [26]. However, PCs can be identified by coexpressing CD146 [13, 14], NG2 [15], *α*SMA [16], and PDGFR-*β* [17] while being negative for PECAM-1 [7]. We observed a remarkable increase of CD146 and NG2 expression on MSCs and depleted-MSCs when cocultured with EPCs. PDGFR-*β* expression decreased but was still the highest expressed gene of all examined PC markers. Analysing the gene expression after different days of coculture, we found the most prominent differential regulation of genes on day 3. The increased CD146 and NG2 expression, the maintained high expression of PDGFR-*β*, and the complete reduction of PECAM-1 in MSCs and depleted-MSCs support the assumption that MSCs and depleted-MSCs differentiate towards a PC-like phenotype.

In addition, the gene expression of depleted-MSCs and MSCs revealed similar results, indicating that CD146 positive cells present in MSCs did not proliferate. This underlines the hypothesis that MSCs and depleted-MSCs can differentiate towards a PC phenotype. In line, immunocytochemistry revealed positive signals of CD146, NG2, and *α*SMA on MSCs which indicates the presence of triple positive cells after 7 days of direct coculture. Interestingly, the immunofluorescence analysis demonstrated a positive signal for *α*SMA at day 7, whereas the mRNA level was already decreased at this time point (data not shown).

We reported the highest upregulation of CD146 and NG2 for MSCs and depleted-MSCs in direct coculture with EPC. Therefore, direct cell-cell contact with EPCs might be an important factor for the differentiation of MSCs and depleted-MSCs towards a PC-like phenotype. The underlying mechanism remains unknown. However, integrins may be involved in this promotion of the angiogenic response of endothelial cells since the *α*6 integrin subunit in MSCs has been reported to play a role in vessel formation and PC differentiation [27].

PCs were originally described to be indistinguishable in culture from MSCs [28]. Covas et al. demonstrated a similar gene expression profile of retinal PCs compared to MSCs from 12 different fetal and adult tissues [13], highlighting the close relationship of both cell populations. Therefore, Covas et al. suggested that MSCs and PCs might be related cells, present at the vascular wall, and constitute a MSC compartment extending throughout the entire organism [13]. The large overall similarity between PCs and MSCs, with regard to their immunophenotype and differentiation potential in vitro, was already described by Shi and Gronthos and Armulik et al. [29, 30]. This supports our hypothesis that MSCs and depleted-MSCs differentiate towards a PC-like phenotype.

Armulik et al. noted that the expression of the combination of markers which were used to confirm the PC phenotype in our study is variable and depends on culture conditions [26]. We used two media conditions based on IMDM. MEM*α* was used for MSCs/depleted-MSCs expansion prior to the experiment. Armulik et al. demonstrated a strong NG2 expression of human placenta derived PCs when cultured in PC growth media (PGM, Promocell); however, staining intensity decreased after transfer to Dulbecco's Modified Eagle Medium (DMEM). In contrast to this finding, *α*SMA staining was enhanced for both human placenta derived PCs and MSCs when cultured in DMEM. This emphasises a crucial, but still unknown, impact of culture conditions.

CD133+ and CD34+ separated cells were highly purified in our study, but these antigens are not expressed on mature endothelial cells [8, 31]. On mRNA level, the investigated EPCs lack the marker of mature endothelial cells PECAM-1 at the beginning. However, lectin positive cells showed a significant increase of PECAM-1 expression. Aguirre et al. [4] reported an upregulation of the endothelial phenotype in EPCs and MSCs after 3 days of direct coculture without any supplementary growth factors. Our data support that EPCs can differentiate into mature ECs by direct cell-cell contact with MSCs. However, in the present study, cocultured EPCs were separated by lectin binding, which favours an additional purification of endothelial cells from a more heterologous EPCs population.

Previous studies showed a positive effect on proliferation and differentiation of endothelial cells treated with growth factors released by PL [23, 32]. Our data support these findings, since PECAM-1 expression of EPCs is higher in IMDM-PL than in IMDM-FCS. Furthermore, we observed that PL diminished the difference in gene expression for all PC genes, in both MSCs/depleted-MSCs and EPCs. PL consists of a composition of growth factors and cytokines, which might be responsible for the reported changes in the gene expression.

Interestingly, PECAM-1 expression seems to be particularly influenced by PL, since PECAM-1 expression is increased in most cell culture conditions in IMDM-PL whereas only in direct cocultures in IMDM-FCS. Therefore, PL might be a trigger for the differentiation of EPCs towards ECs. More recently, a study demonstrated the importance of PDGF and EGF signalling in controlling PC recruitment to luminal tubular structures [33]. Although we have not explicitly examined these signalling molecules in the present study, future work can determine if PL influences these pathways in the same way.

## 5. Conclusion

In summary, we report preliminary data on the effect of cell-cell contact of bone marrow derived MSCs and EPCs with regard to the induction of PC-like cells. The upregulation of several PC marker genes (CD146, NG2, and *α*SMA) indicates the differentiation of MSCs/depleted-MSCs towards PCs in vitro. The change of phenotype is detectable as early as day 3 of coculture.

Moreover, our data confirm that several growth factors, released by PL, contribute to the differentiation of EPCs towards endothelial cells. The effect of PL on PC induction may be further evaluated in future studies. These findings emphasise the concept of coculture strategies to promote angiogenesis for cell-based tissue engineered bone grafts.

## Supplementary Material

Gene regulation in Depleted-MSCs and EPCs after 3 days of culture in IMDM-FCS. Pericyte marker genes CD146 (A, F), NG2 (B, G), *α*SMA (C, H), and PDGFR-*β* (D, I) and endothelial marker gene PECAM-1 (E, J) were detected in depleted-MSCs and EPCs after 3 days in transwell culture, direct coculture and single culture in IMDM-FCS (IMDM supplemented with 10% FCS). Results are presented for each gene as relative change in gene expression (2^-ΔΔCt^) over time between day 0 and day 3 for transwell culture, direct co-culture, and single culture in 5 independent experiments (donor 1-5), and as mean ± error of the mean of all experiments.
Gene regulation in Depleted-MSCs and EPCs after 3 days of culture in IMDM-PL. Pericyte marker genes CD146 (A, F), NG2 (B, G), *α*SMA (C, H), and PDGFR-*β* (D, I) and endothelial marker gene PECAM-1 (E, J) were detected in depleted-MSCs and EPCs after 3 days in transwell culture, direct coculture and single culture in IMDM-PL (IMDM supplemented with 5% FCS, 5% PL). Results are presented for each gene as relative change in gene expression (2^-ΔΔCt^) over time between day 0 and day 3 for transwell culture, direct coculture, and single culture in 5 independent experiments (donor 1-5) and as mean ± standard error of the mean of all experiments.
Gene regulation in MSCs, Depleted-MSCs and corresponding EPCs after 3 days of culture in IMDM-FCS and IMDM-PL. Pericyte marker genes CD146, NG2, *α*SMA, and PDGFR-*β*, and endothelial marker gene PECAM-1 were detected in MSCs, depleted-MSCs and corresponding EPCs after 3 days in transwell culture, direct coculture and single culture in IMDM-FCS (IMDM supplemented with 10% FCS) and IMDM-PL (IMDM supplemented with 5% FCS, 5% PL). Results are presented for each gene as relative change in gene expression (2^-ΔΔCt^) over time between day 0 and day 3 for transwell culture, coculture, and single culture as mean ± standard error of the mean of 5 independent experiments.
