# Supplementary figures and images for: Direct Cell-Cell Contact between Mesenchymal Stem Cells and Endothelial Progenitor Cells Induces a Pericyte-Like Phenotype In Vitro
Source: Biomed Res Int. 2014 Jan 20;2014:395781. doi: 10.1155/2014/395781 (PMC3915932; doi:10.1155/2014/395781)

# depleted-MSK in IMDM-FCS

# EPC in IMDM-FCS

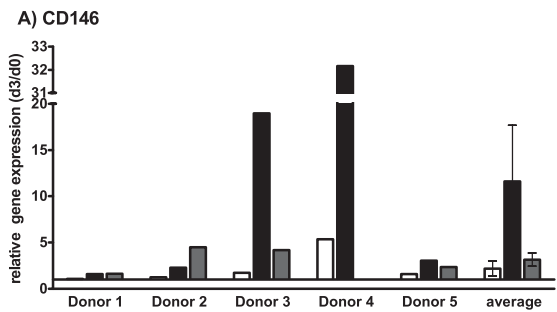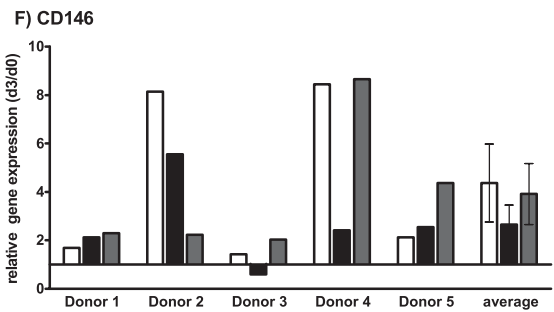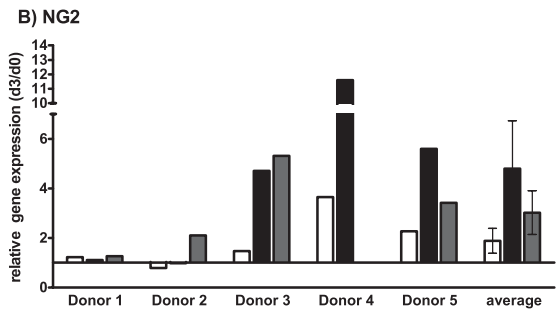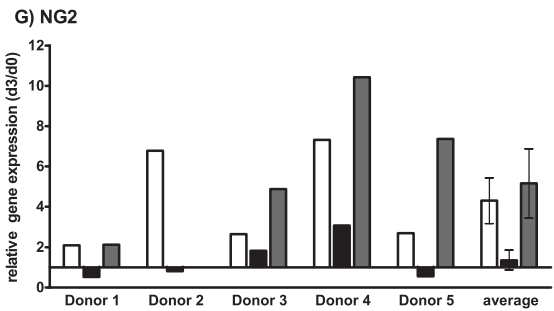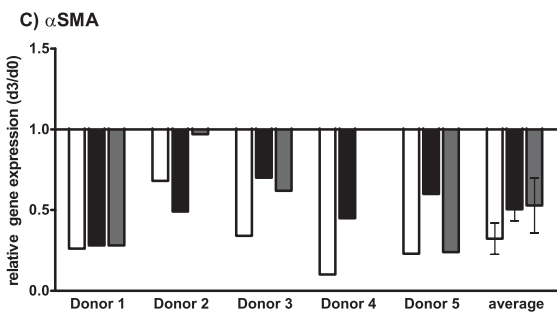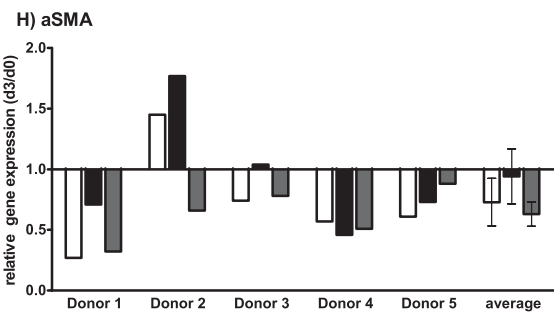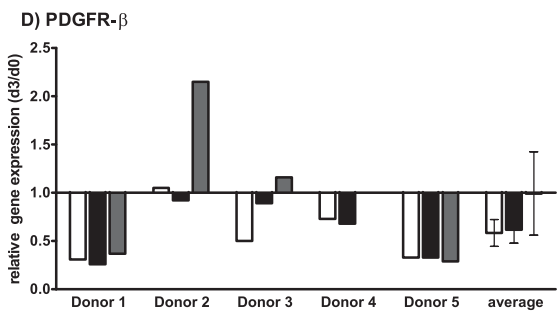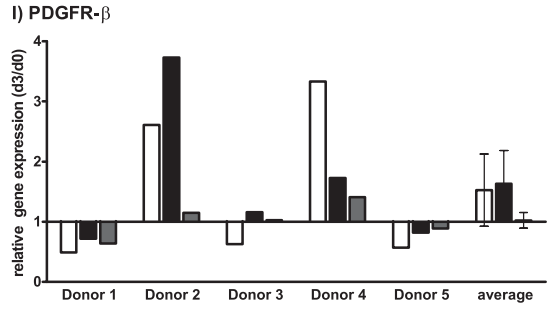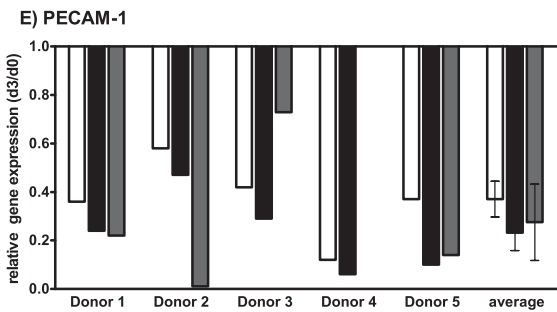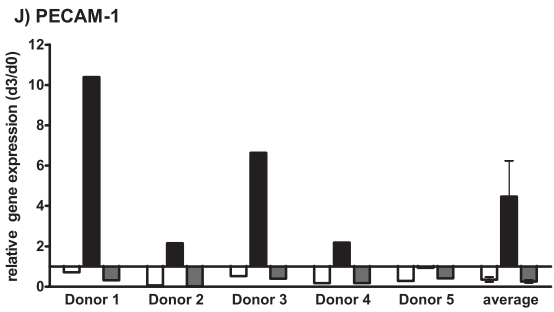

transwell direct co-culture single

Supplement: Supplementary file 1 — Gene regulation in Depleted-MSCs and EPCs after 3 days of culture in IMDM-FCS. Pericyte marker genes CD146 (A, F), NG2 (B, G), αSMA (C, H), and PDGFR-β (D, I) and endothelial marker gene PECAM-1 (E, J) were detected in depleted-MSCs and EPCs after 3 days in transwell culture, direct coculture and single culture in IMDM-FCS (IMDM supplemented with 10% FCS). Results are presented for each gene as relative change in gene expression (2-ΔΔCt) over time between day 0 and day 3 for transwell culture, direct co-culture, and single culture in 5 independent experiments (donor 1-5), and as mean ± error of the mean of all experiments. Gene regulation in Depleted-MSCs and EPCs after 3 days of culture in IMDM-PL. Pericyte marker genes CD146 (A, F), NG2 (B, G), αSMA (C, H), and PDGFR-β (D, I) and endothelial marker gene PECAM-1 (E, J) were detected in depleted-MSCs and EPCs after 3 days in transwell culture, direct coculture and single culture in IMDM-PL (IMDM supplemented with 5% FCS, 5% PL). Results are presented for each gene as relative change in gene expression (2-ΔΔCt) over time between day 0 and day 3 for transwell culture, direct coculture, and single culture in 5 independent experiments (donor 1-5) and as mean ± standard error of the mean of all experiments. Gene regulation in MSCs, Depleted-MSCs and corresponding EPCs after 3 days of culture in IMDM-FCS and IMDM-PL. Pericyte marker genes CD146, NG2, αSMA, and PDGFR-β, and endothelial marker gene PECAM-1 were detected in MSCs, depleted-MSCs and corresponding EPCs after 3 days in transwell culture, direct coculture and single culture in IMDM-FCS (IMDM supplemented with 10% FCS) and IMDM-PL (IMDM supplemented with 5% FCS, 5% PL). Results are presented for each gene as relative change in gene expression (2-ΔΔCt) over time between day 0 and day 3 for transwell culture, coculture, and single culture as mean ± standard error of the mean of 5 independent experiments. [file 395781.f1.pdf]

# depleted-MSC in IMDM-PL

# EPC in IMDM-PL

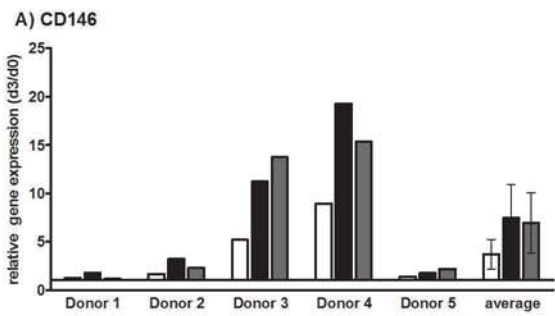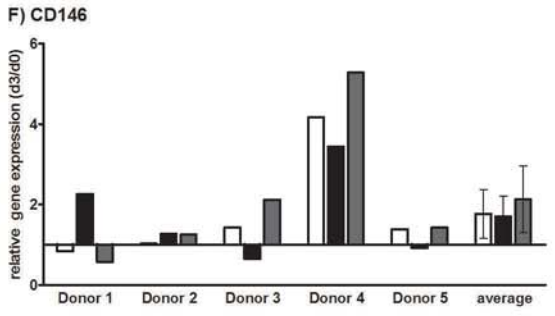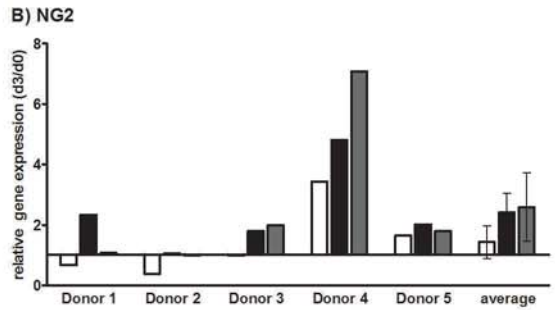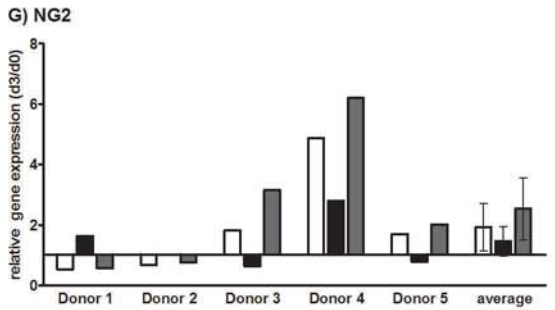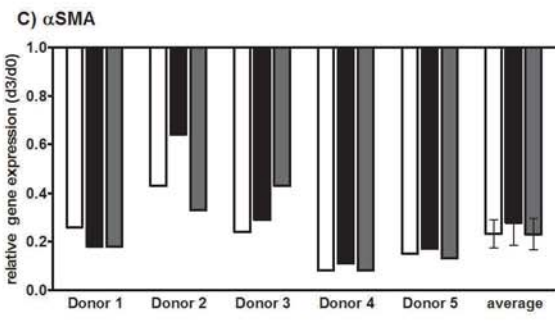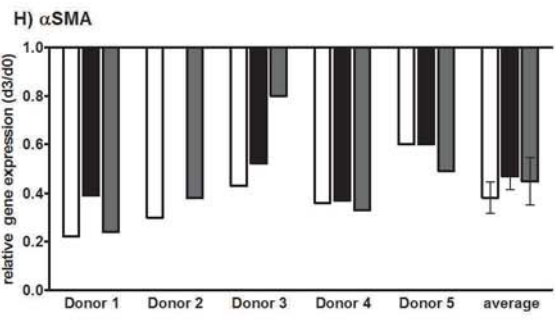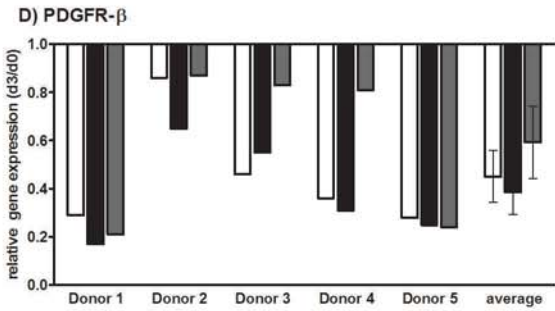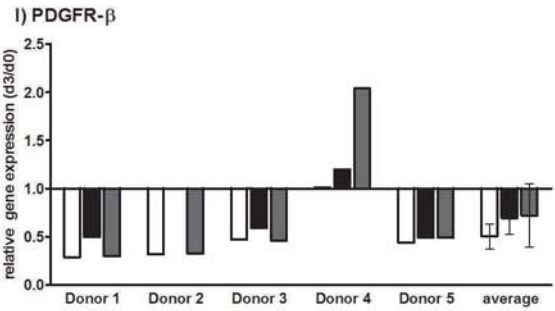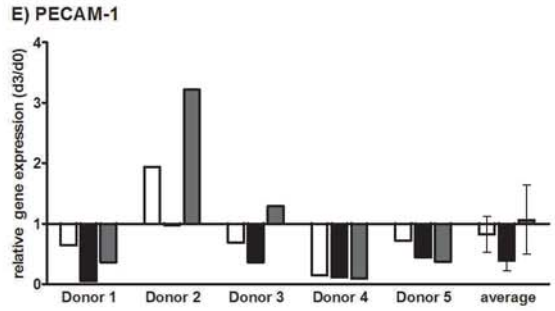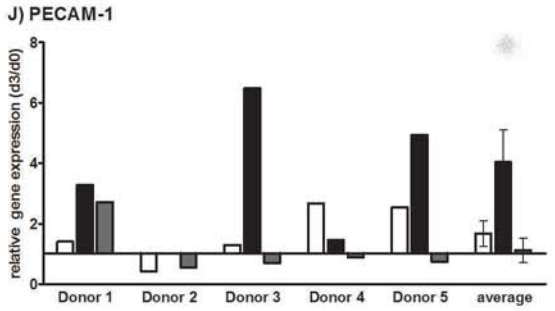

Supplement: Supplementary file 2 [file 395781.f2.pdf]
