# Supplementary material for: Direct Cell-Cell Contact between Mesenchymal Stem Cells and Endothelial Progenitor Cells Induces a Pericyte-Like Phenotype In Vitro
Source: Biomed Res Int. 2014 Jan 20;2014:395781. doi: 10.1155/2014/395781 (PMC3915932; doi:10.1155/2014/395781)
Supplement: Supplementary file 3 [file 395781.f3.pdf]

Gene	Cell type	Condition	IMDM-FCS	IMDM-PL
CD146	MSC	transwell	1.97 (± 0.58)	3.47 (± 0.98)
		direct	15.15 (± 6.99)	8.44 (± 3.50)
		single	3.25 (± 0.89)	4.30 (± 1.62)
	EPC (MSC)	transwell	3.37 (± 1.27)	3.00 (± 0.94)
		direct	2.42 (± 0.78)	1.70 (± 0.51)
		single	3.92 (± 1.26)	2.13 (± 0.82)
	depleted-MSC	transwell	2.19 (± 0.80)	3.69 (± 1.51)
		direct	11.62 (± 6.07)	7.44 (± 3.44)
		single	3.16 (± 0.70)	6.93 (± 3.11)
	EPC (depleted-MSC)	transwell	4.37 (± 1.61)	1.77 (± 0.61)
		direct	2.65 (± 0.81)	1.70 (± 0.51)
		single	3.92 (± 1.26)	2.13 (± 0.82)
NG2	MSC	transwell	2.06 (± 0.21)	1.51 (± 0.34)
		direct	6.40 (± 2.60)	3.11 (± 0.64)
		single	3.21 (± 0.36)	2.32 (± 0.55)
	EPC (MSC)	transwell	3.73 (± 1.32)	3.22 (± 1.48)
		direct	1.86 (± 0.73)	1.36 (± 0.48)
		single	5.16 (± 1.72)	2.54 (± 1.03)
	depleted-MSC	transwell	1.88 (± 0.50)	1.42 (± 0.55)
		direct	4.80 (± 1.94)	2.41 (± 0.64)
		single	3.02 (± 0.88)	2.59 (± 1.14)
	EPC (depleted-MSC)	transwell	4.30 (± 1.13)	1.92 (± 0.78)
		direct	1.36 (± 0.49)	1.46 (± 0.50)
		single	5.16 (± 1.72)	2.54 (± 1.03)
αSMA	MSC	transwell	0.28 (± 0.07)	0.22 (± 0.05)
		direct	0.34 (± 0.09)	0.24 (± 0.05)
		single	0.30 (± 0.06)	0.19 (± 0.04)
	EPC (MSC)	transwell	0.58 (± 0.07)	0.70 (± 0.29)
		direct	0.97 (± 0.20)	0.60 (± 0.10)
		single	0.63 (± 0.10)	0.45 (± 0.10)
	depleted-MSC	transwell	0.32 (± 0.10)	0.23 (± 0.06)
		direct	0.50 (± 0.07)	0.28 (± 0.10)
		single	0.53 (± 0.17)	0.23 (± 0.07)
	EPC (depleted-MSC)	transwell	0.73 (± 0.20)	0.38 (± 0.06)
		direct	0.94 (± 0.23)	0.47 (± 0.05)
		single	0.63 (± 0.10)	0.45 (± 0.10)
PDGFR-β	MSC	transwell	0.51 (± 0.08)	0.38 (± 0.05)
		direct	0.60 (± 0.10)	0.39 (± 0.04)
		single	0.44 (± 0.07)	0.37 (± 0.07)
	EPC (MSC)	transwell	1.35 (± 0.65)	0.70 (± 0.15)
		direct	1.46 (± 0.27)	0.96 (± 0.15)
		single	1.02 (± 0.13)	0.72 (± 0.33)
	depleted-MSC	transwell	0.58 (± 0.14)	0.28 (± 0.00)
		direct	0.62 (± 0.14)	0.39 (± 0.09)
		single	0.99 (± 0.43)	0.59 (± 0.15)
	EPC (depleted-MSC)	transwell	1.53 (± 0.60)	0.51 (± 0.13)
		direct	1.63 (± 0.55)	0.70 (± 0.17)
		single	1.02 (± 0.13)	0.72 (± 0.33)
PECAM-1	MSC	transwell	0.28 (± 0.09)	0.45 (± 0.11)
		direct	0.17 (± 0.05)	0.35 (± 0.14)
		single	0.22 (± 0.05)	0.22 (± 0.06)
	EPC (MSC)	transwell	0.21 (± 0.09)	2.53 (± 0.89)
		direct	8.16 (± 3.49)	10.24 (± 5.94)
		single	0.26 (± 0.08)	1.12 (± 0.40)
	depleted-MSC	transwell	0.37 (± 0.07)	0.83 (± 0.30)
		direct	0.23 (± 0.07)	0.39 (± 0.17)
		single	0.28 (± 0.16)	1.07 (± 0.58)
	EPC (depleted-MSC)	transwell	0.35 (± 0.12)	1.67 (± 0.42)
		direct	4.46 (± 1.77)	4.04 (± 1.08)
		single	0.26 (± 0.08)	1.12 (± 0.40)
